# Hazards of lunar surface exploration: determining the immunogenicity/allergenicity of lunar dust

**DOI:** 10.3389/fimmu.2025.1539163

**Published:** 2025-05-08

**Authors:** Audrie A. Colorado, Cody L. Gutierrez, Mayra Nelman-Gonzalez, Gailen D. Marshall, J. Torin Mccoy, Brian E. Crucian

**Affiliations:** ^1^ KBR, Johnson Space Center Immunology Laboratory, Houston, TX, United States; ^2^ JES Tech, Johnson Space Center Immunology Laboratory, Houston, TX, United States; ^3^ Department of Medicine, The University of Mississippi Medical Center, Jackson, MS, United States; ^4^ National Aeronautics and Space Administration (NASA) Johnson Space Center, Houston, TX, United States

**Keywords:** lunar dust, spaceflight, allergy, immunology, NASA

## Abstract

Although infrequent, there have been Apollo program reports of lunar dust exposure leading to notable upper respiratory symptoms in select crewmembers. Possible mechanisms include particulate irritation, inflammation from toxic insult, or legitimate adaptive immune-mediated response. Although sterile non-protein matter would not be expected to be immunogenic, one Apollo flight surgeon reported increasing symptoms upon repeated perceived exposure with associated eosinophilia, suggestive of possible allergic reactions. Many International Space Station (ISS) crews display a pattern of persistent immune system dysregulation and latent virus reactivation. Some ISS crews manifest atypical respiratory and/or dermatitis symptoms which could have an allergic component. It is logical to anticipate crew immune dysregulation could worsen during prolonged deep space missions and planetary surface hazards will only complicate crew health risks. Allergic (i.e. mast cell-mediated) reactivity could adversely increase negative clinical and operational impacts for long-duration lunar astronauts and affect countermeasure requirements for surface vehicles. This study investigated whether lunar dust exposure could possibly elicit an IgE mediated allergic response during spaceflight by utilizing in vitro cell culture models. Our laboratory was officially approved for receipt of actual lunar dust samples from the Apollo 16 mission from NASA. These samples were used to complete the proposed set of in vitro cell culture experiments, using human peripheral blood mononuclear cells (PBMC) from healthy individuals, and basophils and eosinophil cell lines. Cells were co-cultured with cellular mitogens, common recall antigens (Der p1), fine ground silica quartz (control), or lunar dust, to study whether lunar dust exposure could alter the generation of selective immune responses associated with clinical allergic reactions. Measured outputs included supernatant-derived total IgE, tryptase, histamine, and selected cytokine levels. Cellular activation was monitored by assessing activation markers via flow cytometry. EM/x-ray analysis was used to determine cellular interactions with dust particles. The assessments in primary human blood immune cells indicated no evidence for cellular responsiveness nor ’allergy-like‘ reactivity to lunar dust. Assessments using purified ’allergic‘ cell lines, did yield some unique but mild responsiveness to lunar dust, however such cells lines can have response profiles somewhat different from their in vivo counterparts. This study determining the allergy specific immune responses, will help guide NASA to develop mitigation techniques and potential countermeasures necessary in the event of excessive exposure to lunar dust during lunar surface EVAs.

## 1 Introduction

Lunar dust is a unique material that consists of very sharp micron-scale particles that stick to skin, clothing, and equipment, and are easily inhaled (1). Crew members and landing vehicles will inevitably be exposed to lunar dust in future lunar missions. While much is known of the toxic hazard potential, some gaps are present in the evidence base. During the Apollo moon missions, there were consistent reports of lunar dust exposure, with this exposure sometimes leading to upper respiratory symptoms in both astronauts and ground support personnel (2). There could be several mechanisms by which lunar dust causes symptoms, including simple particulate irritation of mucus membranes, the dust acting as a toxin, or by activating the immune system to demonstrate one or more inflammatory reactions, some of which could be allergic in nature. International Space Station (ISS) crews have displayed a pattern of immune system dysregulation that predispose them to both inflammation and allergy-like respiratory and cutaneous symptoms (3–5). The potential for lunar dust to generate immediate sensitivity reactions that could impact mission operations remains uninvestigated.

This study directly addresses whether actual lunar dust elicits an immunogenic response. Our laboratory received lunar dust acquired from the Apollo 16 mission from soil and rake sample collections from surface regolith of the ejector blanket of South Ray Crater at Station 8 (6). A previous JSC animal lunar dust inhalation study examined direct lung toxicology and inflammatory responses but did not assess any allergy-related sensitizations or reactions (7). The area of focus for this study is the unique aspect of dust exposure related to development of an immune milieu indicative of increased allergic sensitization of reactivity. As previously detailed, there are credible anecdotal reports from the Apollo program of both crewmembers and flight surgeons experiencing what were described as ‘allergic’ (upper respiratory) symptoms. One surgeon documented increasing symptoms with repeat perceived exposure, and documented eosinophilia which can be observed during more severe allergic reactions. However, there is currently only anecdotal data regarding allergy risk and lunar dust exposure. The data from this study will help inform how NASA responds if an astronaut experiences upper respiratory symptoms on the lunar surface. The data will also potentially influence operations and/or vehicle engineering design for dust containment. The goal of this study is to address, in a simple cell culture experiment, whether lunar dust can act as an allergen, an adjuvant, or a cellular toxin.

## 2 Methods

### 2.1 Human subjects

The conducted work met all federal and local requirements for human subjects’ protection and complies with the NASA Policy Directive (NPD) 7100.8E “Protection of Human Research Subjects”. The Immunology laboratory at Johnson Space Center (JSC) has performed numerous ground and flight studies using human test subjects, with the review and approval of the local Institutional Review Board (IRB). All study participants were provided informed written consent prior to participation.

Subjects were active, healthy test subjects enrolled and screened by the NASA JSC Test Subject Facility (TSF). A total of 6 subjects were recruited (5 M, 1 F). Subject solicitation was mediated by the TSF and informed consent was obtained by the PI. Institutional review board approval was obtained from the Committee for the Protection of Human Subjects at the JSC; Houston, TX. Subject confidentiality is maintained, and data will only be publicly disclosed in a summarized, nonidentifiable fashion.

### 2.2 Cell lines

Basophil cell line, KU812 (ATCC, USA), was maintained as recommended in RPMI media + 2mM glutamine (Gibco, Rockville, MD, USA) supplemented with 10% fetal bovine serum (ATCC, USA). Cultures used 1e6 cells/mL cocultured with mitogens and recall antigens for 48 hours. All cells were cultured at 37°C, 95% humidity and 5% CO_2_.

Eosinophil cell line, Eol-1 (Millipore Sigma, Burlington, MA, USA), was maintained as recommended in RPMI media + 2mM glutamine supplemented with 10% fetal bovine serum. In preparation of experimental setup, cells were treated with 5μM butyrate (Sigma, St. Louis, MA, USA) for 7 days to induce differentiation of mature eosinophils (**Supplementary Figure S1**). Cultures used 1e6 cells/mL cocultured with mitogens and recall antigens for 48 hours. All cells were cultured at 37°C, 95% humidity and 5% CO_2_.

### 2.3 Human donor primary cell cultures

For this study, peripheral blood samples were collected in lithium heparin blood tubes (Greiner BioOne, Monroe, NC, USA) by venipuncture. All live cell culture assays were performed using whole blood or isolated peripheral blood mononuclear cells (PBMCs), which were purified by Ficoll (Cytiva, Marlborough, MA, USA) density-gradient centrifugation as previously described (3). The PBMC cultures were performed using 1e6 PBMCs/mL and whole blood cultures used 100μL/mL culture media.

### 2.4 Culture stimulations

Lunar dust from Apollo 16 missions was procured from NASA Johnson Space Center through the official “Request for Lunar Dust Samples for Biomedical Research” process. Through preliminary work, and per the supplying NASA archivist statements, the variability in size of the dust (sample ID 68501) made it difficult to precisely refine and weigh less than 1mg of dust of the appropriately sized particles for testing. Essentially, the particles received were of great varying size, below their ability to refine. To ensure adequate cell interaction with the dust for downstream assays, a decision was made to use approximately 2mg total weight of lunar dust mixed with 1mL of culture media per culture test. There was no apparent bacterial or yeast growth in the culture media following incubation up to 14 days during protocol development.

Pure, fine ground (5μM) silica was cultured with cells at 100μg/mL culture (U.S. Silica, Katy, TX, USA). Silica is a standard simulant used by the National Institutes of Health to study respiratory system toxicity as it is known to cause silicosis and was used as a terrestrial control to lunar dust in this study similar to the previous NASA animal lunar dust inhalation toxicity study (7, 8).

The common house dust mite allergen, *Dermatophagoides pteronyssinus* allergen 1 (nDerp1) (9), was used to stimulate cultures for the recall antigenic control (InBio, Charlottesville, VA, USA). Natural Derp1 was activated before each test using 1mM Dithiothreitol to regenerate its thiol group, which becomes oxidized during purification. Activated nDerp1 was cultured with cells at 1μg/mL.

Mitogen stimulation cultures, as a stimulatory positive control to induce activation, used 0.125 μg/mL anti-CD3 and 0.05 μg/mL anti-CD28 soluble antibodies (both Cytek Biosciences, Freemont, CA, USA), or 10 μg/mL of Staphylococcus enterotoxin B (SEB) (Sigma-Aldrich, St Louis, MO, USA).

### 2.5 Flow cytometry

Flow cytometry was performed on the Northern Lights (Cytek Biosciences, Freemont, CA, USA) using a panel of antibodies listed in **Supplementary Table S1**.

### 2.6 Proliferation assay

Isolated human PBMCs were stained with CFSE cell division tracking dye according to manufacture instructions (Biolegend, San Diego, CA, USA). Briefly, cells were stained with 1μM CFSE dye in the dark and quenched with 100% FBS followed by multiple PBS washes before culture set up. Stained PBMCs were cultured with mitogens or stimulations and incubated at 37°C, 95% humidity and 5% CO_2_ for 6 days. After culture, the cells were stained with a cell viability stain, CD4 and CD19 antibodies to distinguish cell proliferation in live T and B cells, respectively. Cell division was determined by flow cytometry.

### 2.7 Enzyme linked immunosorbent assays

Supernatants from whole blood cultures were collected after the 48 hour culture times and leukotriene, histamine and IgE were quantified using ELISA kits (Abcam, Cambridge, UK). Supernatants were undiluted to quantify leukotriene B4, diluted 1:50 to quantify histamine, and diluted 1:50 to quantify IgE production. Optical density was measured using the Tecan microplate reader (Tecan, Switzerland). Protein concentration was interpolated using a four parameter logistic algorithm standard curve. Data was analyzed using Prism v10.0 (Graphpad, Boston, MA, USA).

### 2.8 Cytokine detection

Supernatants from whole blood cultures were collected after the 48 hour culture times and cytokine production was determined using the MILLIPLEX 13-plex cytokine array (Millipore Sigma, Chicago, IL, USA). Mean fluorescent intensity of the cytokine bead array was measured and data was generated using the Luminex^®^ 200™, HTS, FLEXMAP 3D^®^, MAGPIX^®^ instrument with xMAP^®^ INTELLIFLEX software (Luminex, Austin, TX, USA). Data was analyzed using Prism v10.0 (Graphpad, Boston, MA, USA).

### 2.9 Microscopy

Samples processed for environmental scanning electron microscopy (ESEM) were fixed following culture using a solution containing 2% glutaraldehyde (Sigma-Aldrich, St. Louis, MO, USA) and 3% formaldehyde (Sigma-Aldrich) in sterile phosphate buffer solution (PBS, pH 7.4, ThermoFisher Scientific, Waltham, MA, USA) for 1 hour at room temperature in the dark, then stored at 4°C. Thefixed solutions were covered in aluminum foil and kept at ambient temperature for 30 minutes. The samples were then stored at 4°C until processing.

Approximately 100mL of fixed sample was loaded onto an Isopore TSTP membrane filter (3.0 μm pore size, 13 mm diameter, Millipore Sigma) and gravity filtered to reduce disturbance to any structure present in the sample. The samples were washed twice with 200μL PBS (ThermoFisher Scientific) then rinsed with 1000μL of filtered, sterile milli-Q water. Samples were then sequentially dehydrated using freshly prepared ethanol (200 proof, Sigma-Aldrich) in filtered, sterile milli-Q water. Initial dehydration involved two sequential 30 minute rinses with 500μL of 25% ethanol then 500μL of 50% ethanol. This process was continued by applying increasing concentrations of ethanol at 60%, 70%, 80%, 90% and 100% for 10–15 minutes each. Following dehydration, the filter was mounted on an aluminum mount flat pin with carbon conductive tab (Electron Microscopy Sciences, EMS, Hatfield, PA, USA). The samples were dried for 24 hour inside a biosafety cabinet, then placed into the ESEM chamber overnight for additional drying under vacuum. Following drying, the samples were sputter coated with 2 nm iridium on a Cressington Sputter Coater 298 HR that uses a Cressington Thickness Controller MTM 20.

Imaging was performed with a ThermoFisher Scientific FEI Quanta 250 using the ESEM mode along with a GAD detector. All images were acquired at 10 kV in back scatter electron mode at a pressure of ~1.4 Torr. EDS analysis was conducted using an Ametek EDS system with an Apollo X/Octane Pro detector and TEAM EDS software.

### 2.10 Statistical analysis

This study incorporated *in vitro* cell culture analysis from primary human subject blood samples for both whole blood and PBMCs as well as cell lines. A total of 6 human subjects were enrolled for this study to analyze T, B, monocyte, eosinophil, and basophil cell activation, and IgE, leukotriene, histamine, and cytokine production. Cell lines used were of eosinophil and basophil lineage and experiments were conducted in replicates of 3. Statistical analysis was performed on each separate output of data. Cellular activation of all cell populations in whole blood or PBMC cultures were analyzed by Two-Way ANOVA with Dunnett’s multiple comparisons test. Cellular activation of eosinophil and basophil cell lines, proliferation data for T and B cells, IgE, leukotriene, histamine, and cytokine production were all analyzed using Ordinary One-Way ANOVA with Dunnett’s multiple comparisons test. P values indicated by asterisks in graphs are as follows: * P<0.05, ** P<0.005, *** P<0.0005, **** P<0.0001.

## 3 Results

### 3.1 Immediate cellular responses

To determine if *in vitro* exposure to lunar dust resulted in immediate mitogenic activation of any subset of human peripheral blood immune cells, both PBMC and whole blood from healthy human test subjects were cultured with mitogens SEB or αCD3/αCD28 (positive controls), Der P1 (common recall antigen), or silica (lunar dust simulant control) in parallel to lunar dust.

Flow cytometric analysis of unstained lunar dust or fine ground silica was first conducted to confirm these particles had unique forward- and side scatter properties and would not interfere with our cellular analysis (**Supplementary Figure S2**). In comparison to whole blood cellular populations this study will focus on (granulocytes, monocytes and lymphocytes), the scatter properties of silica and lunar dust were below the size seen in the cell population gates to be analyzed.

**Figure 1:**
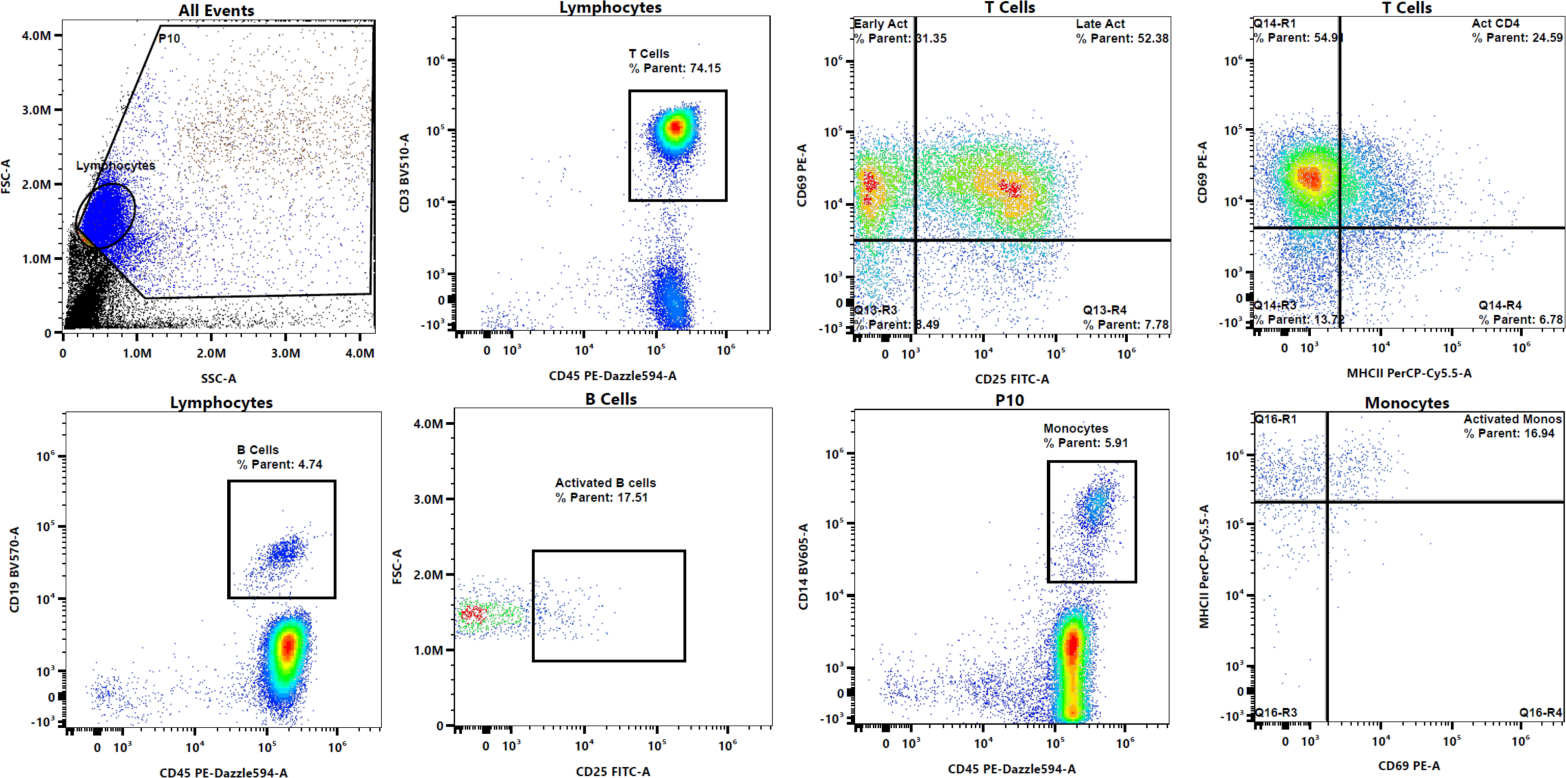
Representative flow plots to depict gating strategy to identify T cells, B cells, and monocytes, and their activation.

**Figure 2:**
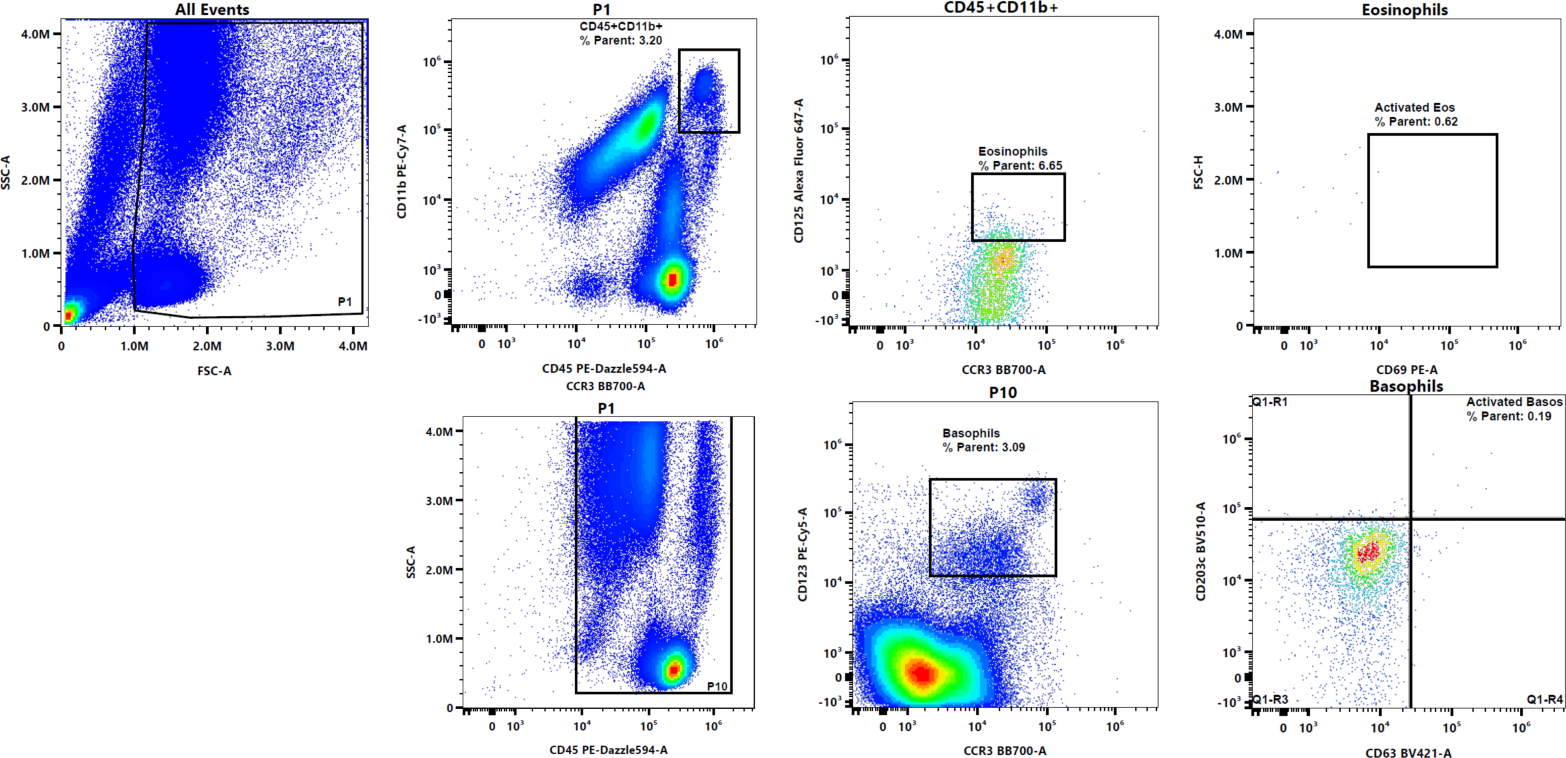
Representative flow plots to depict gating strategy to identify eosinophils and basophils, and their activation.

Cellular activation is vital in the immune response against a foreign invader. It is a cascade of events that leads to the formation of appropriate immune cell responses. Whole blood and PBMC cultures were set up to determine immediate cellular responses including cellular activation of T cells, B cells, monocytes, eosinophils and basophils. Representative flow cytometry plots to show gating strategy of the T cell (CD45+CD3+CD25+CD69+), B cell (CD45+CD19+CD25+), and monocyte (CD45+CD14+MHCII+CD69+) populations in PBMC cultures are displayed in **Figure 1** while activated eosinophil (CD45+CD11b+CCR3+CD125+CD69+) and activated basophil (CD45+CCR3+CD123+CD63+CD203c+) populations in whole blood are displayed in **Figure 2**.

**Figure 3:**
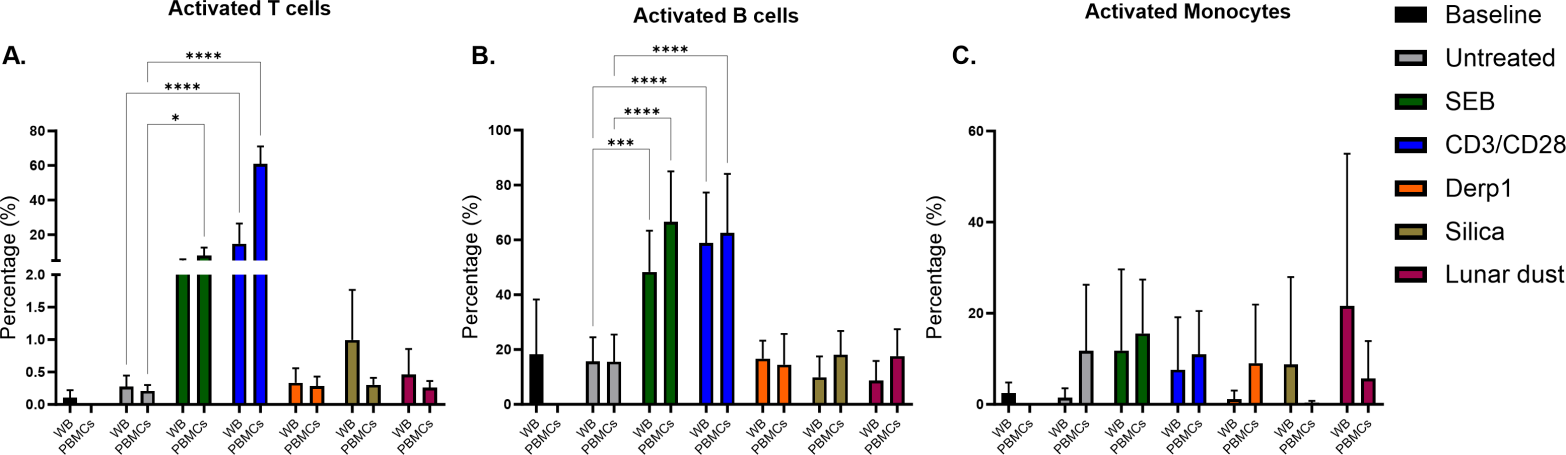
Percentage of **(A)** activated T cells **(B)** activated B cells and **(C)** activated monocytes in whole blood (WB) and PBMC cultures at baseline collection, and after 48 hour stimulation. * P<0.05, *** P<0.0005, **** P<0.0001.

The percentage of T cells in whole blood and PBMC cultures after 48 hours did not change compared to baseline collection and no treatments/stimulations induced any significant changes. The percentage of B cells did significantly decrease in whole blood and PBMC cultures from baseline with the decrease being slightly more in PBMC cultures. However, there were no significant differences among treatment groups compared to untreated control 48 hour culture. The decrease in B cells from baseline after 48 hour culture is expected without specific B cell stimulation and growth factors added (data not shown). It takes over 48 hours for B cells to recover and adapt to culture conditions using the T cell dependent factors in untreated cultures to maintain survival and cell growth (10–12). Relatively little alteration in the relative percentages of these cells was expected given the short-term duration of the cultures. After 48 hours of culture, significant T cell and B cell activation was only seen in the positive control stimuli in comparison to untreated control group (**Figures 3A, B**). These results were expected for the positive controls, and not for the recall antigen, positive simulant silica, or lunar dust, as we hypothesized an allergic response requiring different cell population responses, specifically basophils, mast cells and eosinophils. After 48 hours of culture, there was a significant decrease in the percentage of monocytes in both whole blood and PBMC cultures with αCD3/αCD28, SEB, and silica stimuli, but not with Derp1 or lunar dust indicating cell death in response to those specific stimuli (data not shown). There was no monocyte activation seen in any stimuli group compared to untreated control (**Figure 3C**). These results would suggest monocytes do not play a significant role in the immune response to Derp1, silica or lunar dust stimuli in this model.

**Figure 4:**
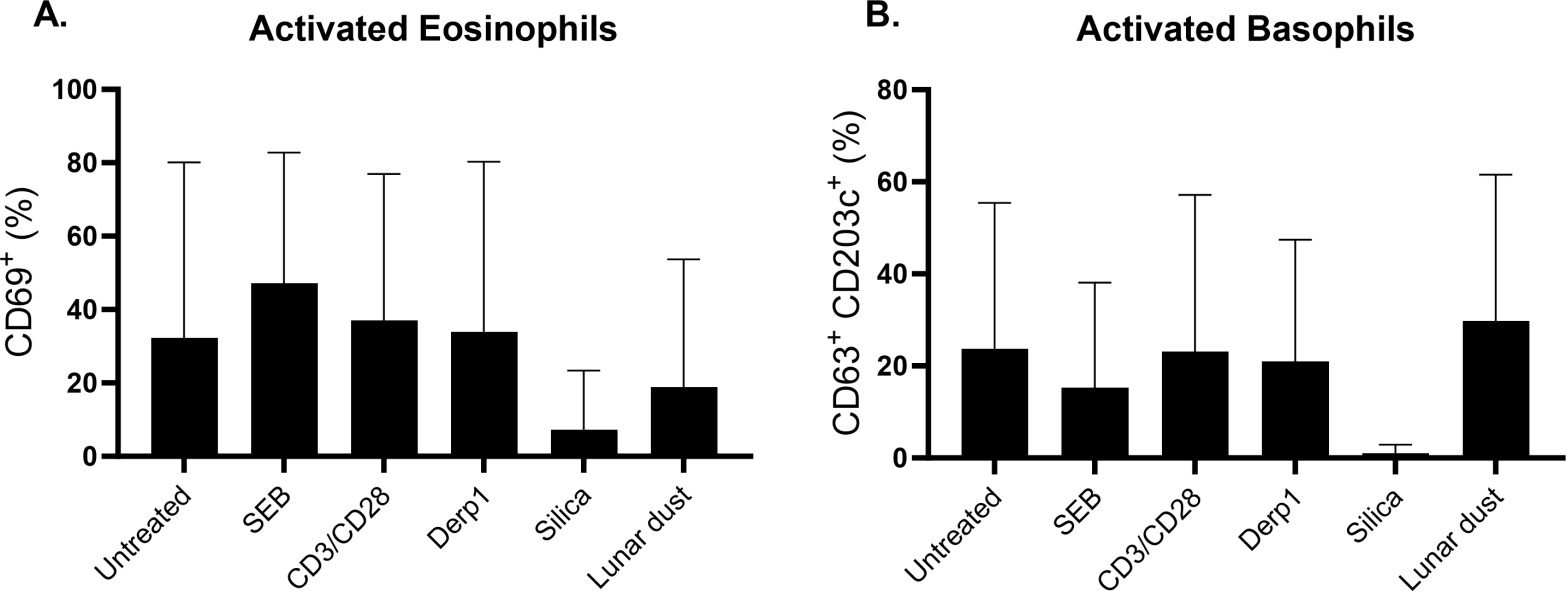
Percentage of **(A)** activated eosinophils and **(B)** activated basophils after 48 hour culture stimulation compared to untreated control.

Eosinophils and basophils are white blood cells found in the granulocyte cell population (**Supplementary Figure S2**). They are involved in immune cell responses to inflammation, parasitic infections, and allergic responses. The roles of eosinophils and basophils includes enzymatic granule production, reactive oxygen species production, antigen presentation, among others (13–16). We focused on these cell populations from primary human donors and cell lines for the potential roles they can play in allergic reactions. We did not see any significant changes in the activation of eosinophils (CD69+) in response to any stimuli in whole blood cultures after 48 hours (**Figure 4A**). Similarly, the activation of basophils (CD203c+CD63+) showed no changes (**Figure 4B**). This could be explained by the diminishment of the CD45+CCR3+CD123+ cell population with the presence of silica most likely due to an exhaustive basophil response.

**Figure 5:**
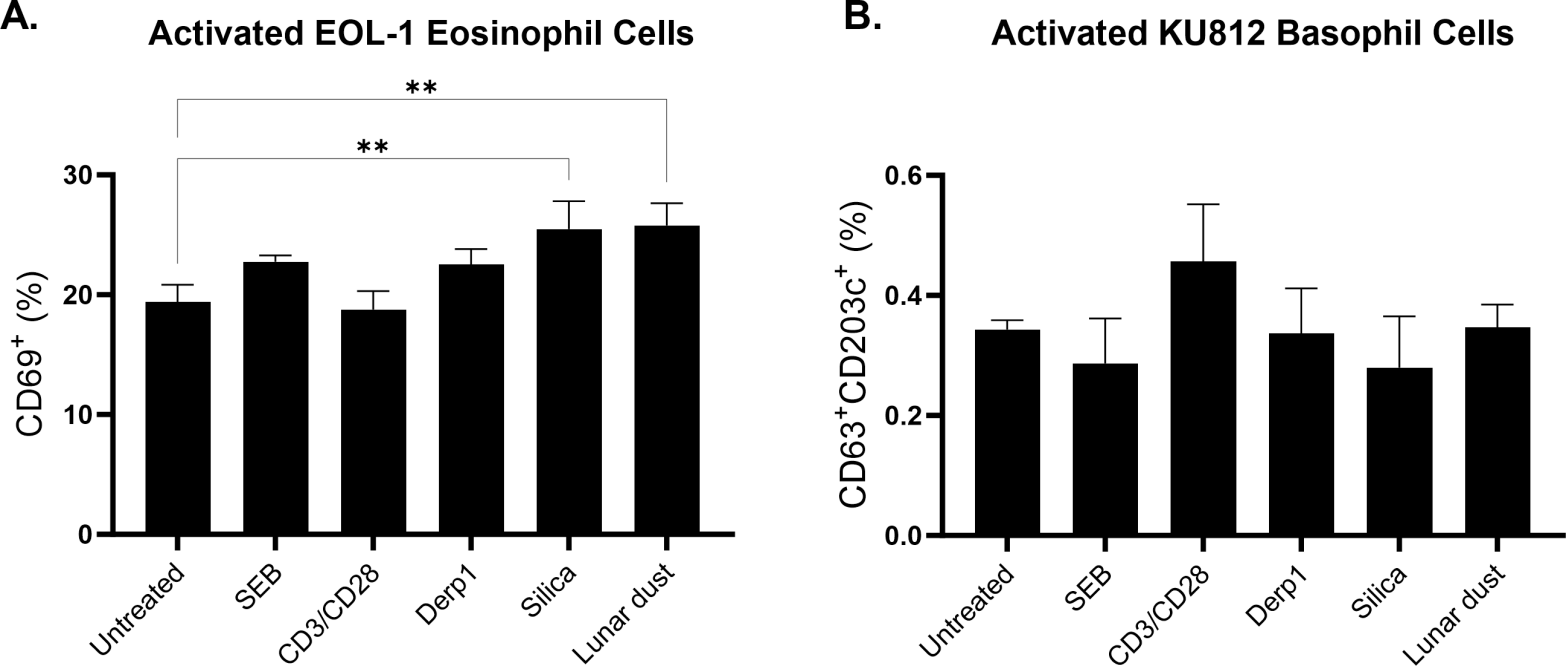
Percentage of activated **(A)** EOL-1 cell line eosinophils and **(B)** KU812 cell line basophils after 48 hour culture stimulation in comparison to untreated control. ** P<0.005.

The same experimental setup was conducted using eosinophil (EOL-1) and basophil (KU812) cell lines. Silica and lunar dust significantly induced eosinophil activation in the cell line cultures after 48 hours of stimulation whereas no stimuli induced a change in the basophil cell line cultures in comparison to the untreated control (**Figures 5A, B**).

**Figure 6:**
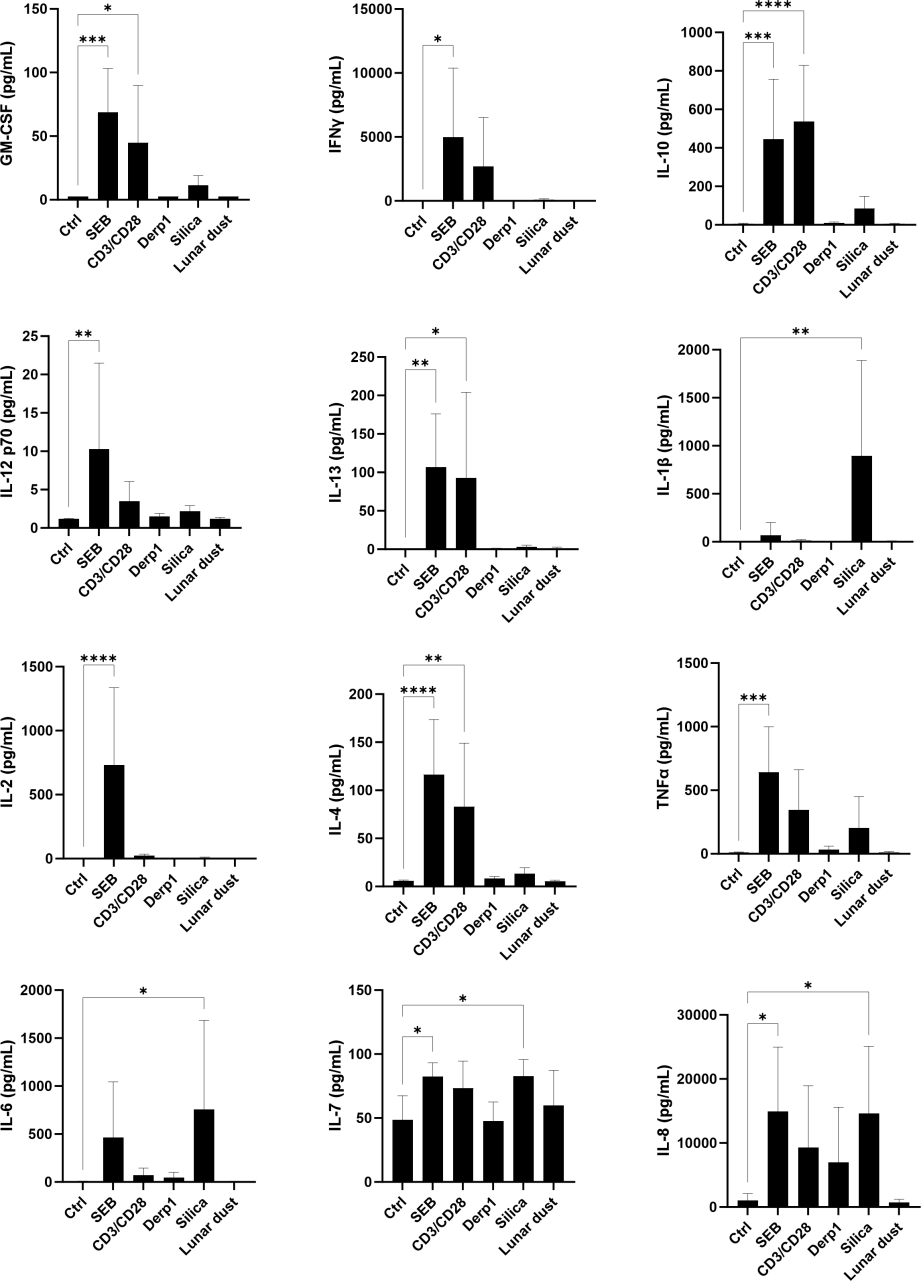
Supernatant cytokine concentration (pg/mL) in whole blood cultures after 48 hour stimulation. * P<0.05, ** P<0.005, *** P<0.0005, **** P<0.0001.

To fully understand the responses seen when lunar dust is introduced to immune cells, this study looked at supernatant derived cytokine production in whole blood cultures after 48 hour stimulation (**Figure 6**). As expected, we saw a significant increase in pro-inflammatory cytokines in response to the positive control mitogens, SEB (GM-CSF, IFNγ, IL-12, IL-2, TNFα, IL-7, IL-8) and the T cell stimuli αCD3/αCD28 (GM-CSF, IL-12). There were also increases seen in some cytokines in response to SEB and αCD3/αCD28 (IL-10, IL-13, IL-4). As expected, silica induced significant increases only in proinflammatory cytokines (IL-1, IL-6, IL-7, IL-8) while lunar dust did not elicit any significant cytokine response. This suggests silica is inducing an innate immune response, but not lunar dust and this could be explained by the small particulate size of silica in comparison to the lunar dust sample used allowing the cell-particulate interaction needed to induce such a response.

Our study focused on whole blood cultures to capture the entire cellular population dynamic during an immune reaction in response to our specific stimuli. The inherent benefits of a whole blood culture system were previously discussed. The limitations of this include interpretation of specific cell types producing these cytokines. However, assumptions can be made on which cell types are producing cytokines based on cell activation data shown earlier. As expected, there were significant increases in cytokines in response to our positive mitogenic control. The absence of cytokine production in response to lunar dust is similar to what was seen in our inhalation study whole blood cultures (17).

**Figure 7:**
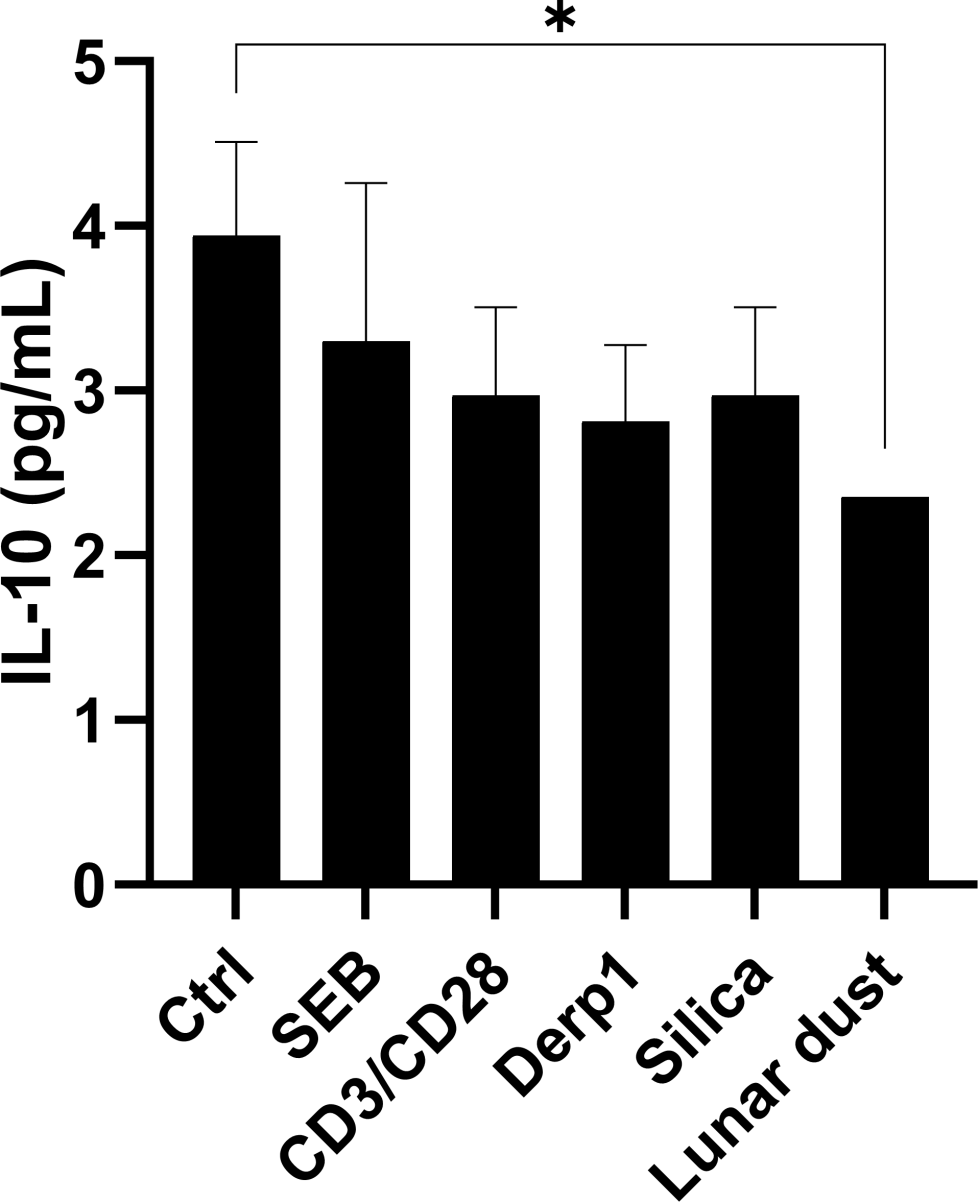
Supernatant cytokine concentration (pg/mL) in EOL-1 eosinophil cell line cultures after 48 hour stimulation. * P<0.05.

**Figure 8:**
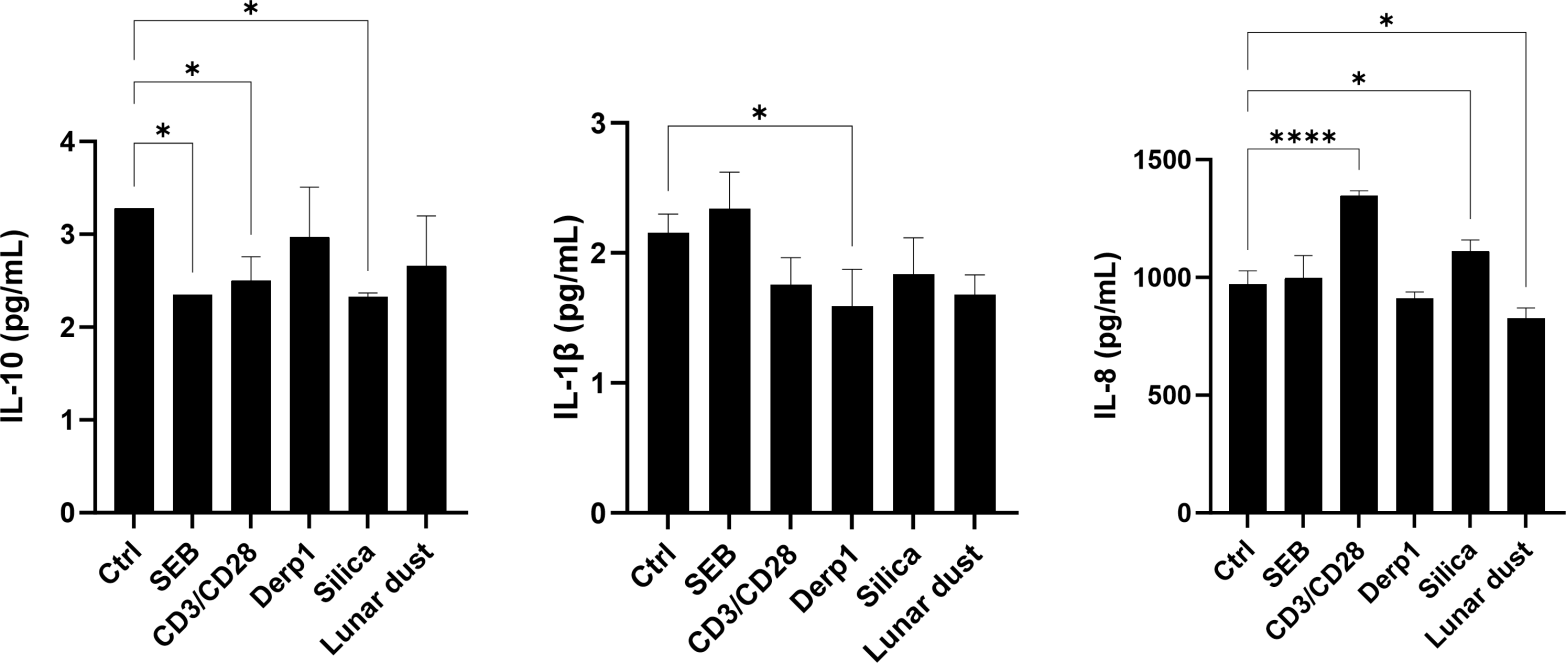
Supernatant cytokine concentration (pg/mL) in KU812 basophil cell line cultures after 48 hour stimulation. * P<0.05, **** P<0.0001.

This study also looked at eosinophil cell line cultures (EOL-1) and basophil cell line cultures (KU812) for direct cell specific cytokine responses after 48 hours of culture stimulation as these cell types are key players in allergic nasal reactions. There was only a significant decrease in the anti-inflammatory cytokine IL-10 in response to lunar dust in the eosinophil cells, although eosinophils are not a major source of IL-10 in allergic pathophysiology (**Figure 7**). In the basophil cell line, we detected mostly decreases in cytokines in response to SEB, αCD3/αCD28, and silica (IL-10), Derp1 (IL-1β), and lunar dust (IL-8). Increases were also seen in response to αCD3/αCD28 and silica (IL-8) (**Figure 8**).

**Figure 9:**
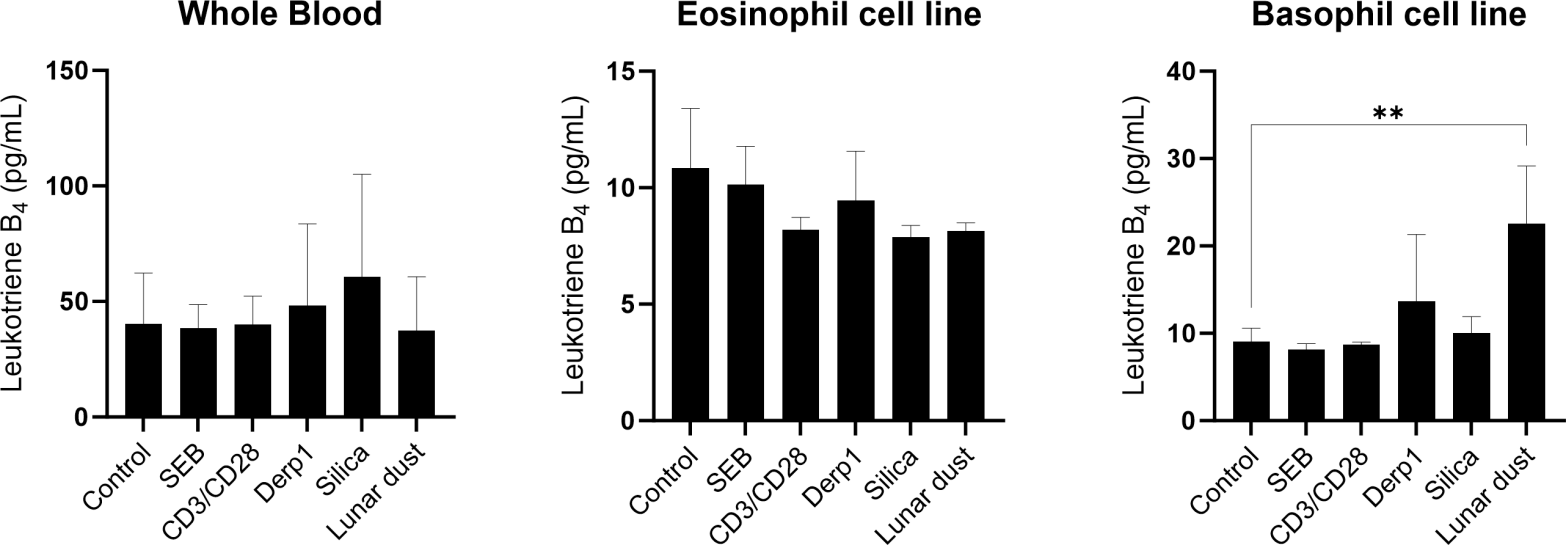
Leukotriene B4 concentration (pg/mL) in whole blood, eosinophil, or basophil cultures after 48 hours stimulation. ** P<0.005.

**Figure 10:**
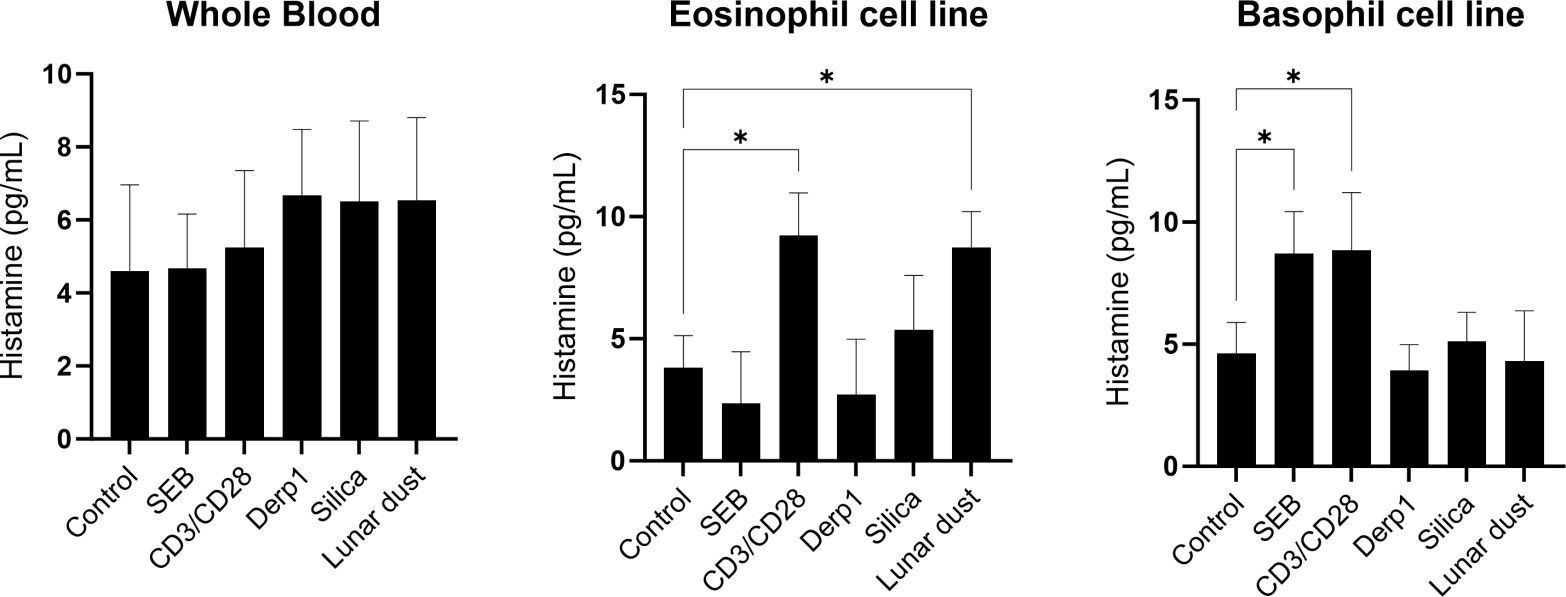
Histamine concentration (pg/mL) in whole blood, eosinophil, or basophil cultures after 48 hours stimulation. * P<0.05.

**Figure 11:**
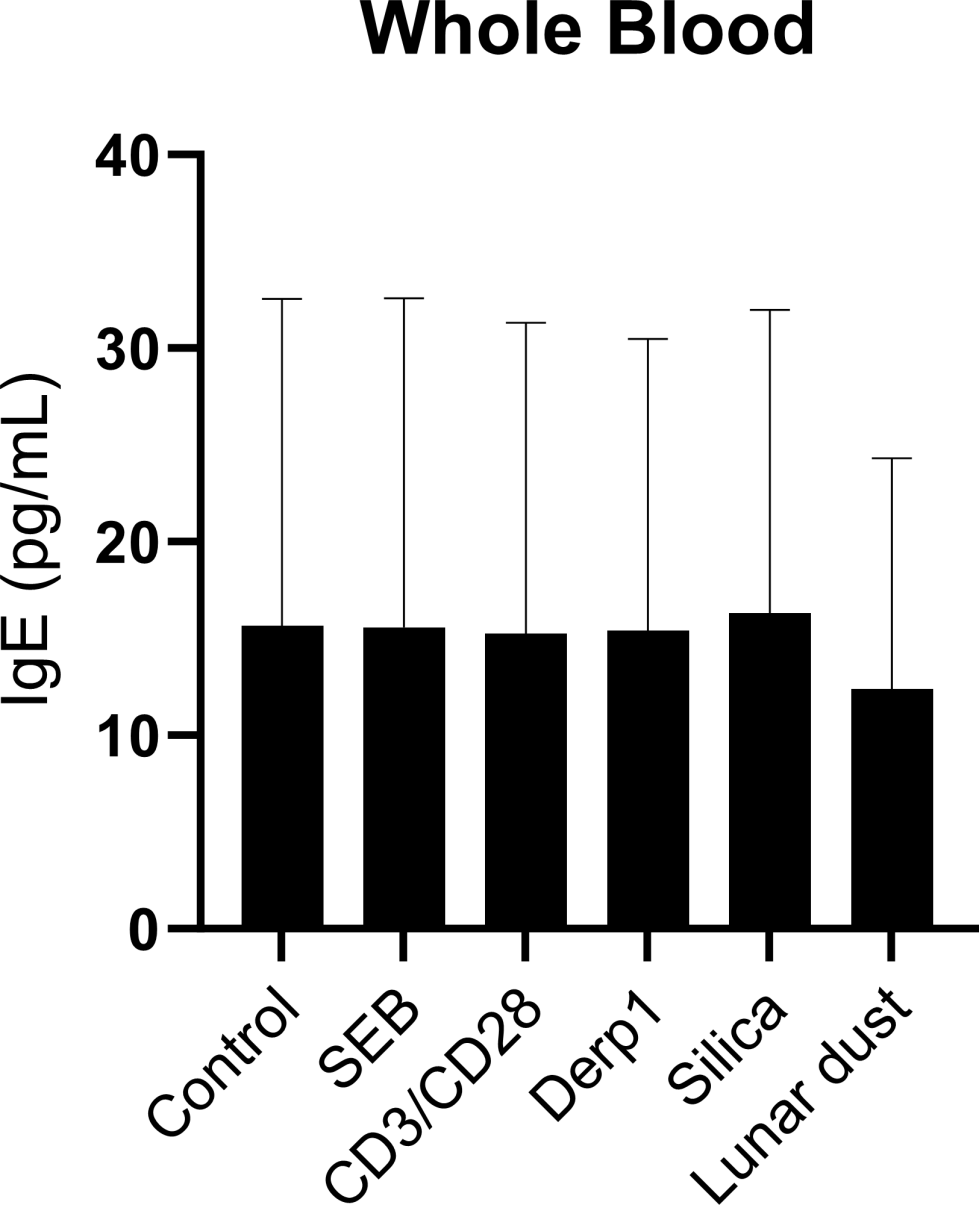
IgE concentration (pg/mL) in whole blood cultures after 48 hours stimulation per subject.

Elevated levels of IgE in whole blood can be indicative of allergies. To determine if subjects responded to lunar dust, *in vitro* IgE response using whole blood was utilized. This study investigated the allergic responses to lunar dust specifically in supernatant derived histamine, leukotriene B4 and IgE from 48 hour stimulated cultures of whole blood, eosinophil cell line and basophil cell line. Leukotriene B4 production was significantly increased by the basophil cell line in response to lunar dust (**Figure 9**). Furthermore, lunar dust exposure induced histamine production in the eosinophil cell line (**Figure 10**). As eosinophils are not producers of histamine, this could be attributed to a percentage of undifferentiated myeloblasts in the EOL-1 cell suspension. Interestingly, there was no significant response to any stimuli, including lunar dust, in total IgE production in whole blood, eosinophil, or basophil cultures (data not shown). This data suggests lunar dust can induce a potentially proallergic milieu in specific purified artificial cell lines representing human basophils and eosinophils. Although, no responses were seen in primary whole blood cultures, this could be explained by the counteractive responses of other immune cells in these cocultures. The human whole blood cultures presented a wide range of IgE concentration per subject which is well known regardless of the atopic status on the individual. There were no significant changes in total IgE production in response to any stimuli, but data is graphed per subject to reveal the extent of the breadth of data collected and the necessity for an increase in human subject enrollment with specific medical history information such as allergies and antihistamine use documented (**Figure 11**).

### 3.2 Cell proliferation

**Figure 12:**
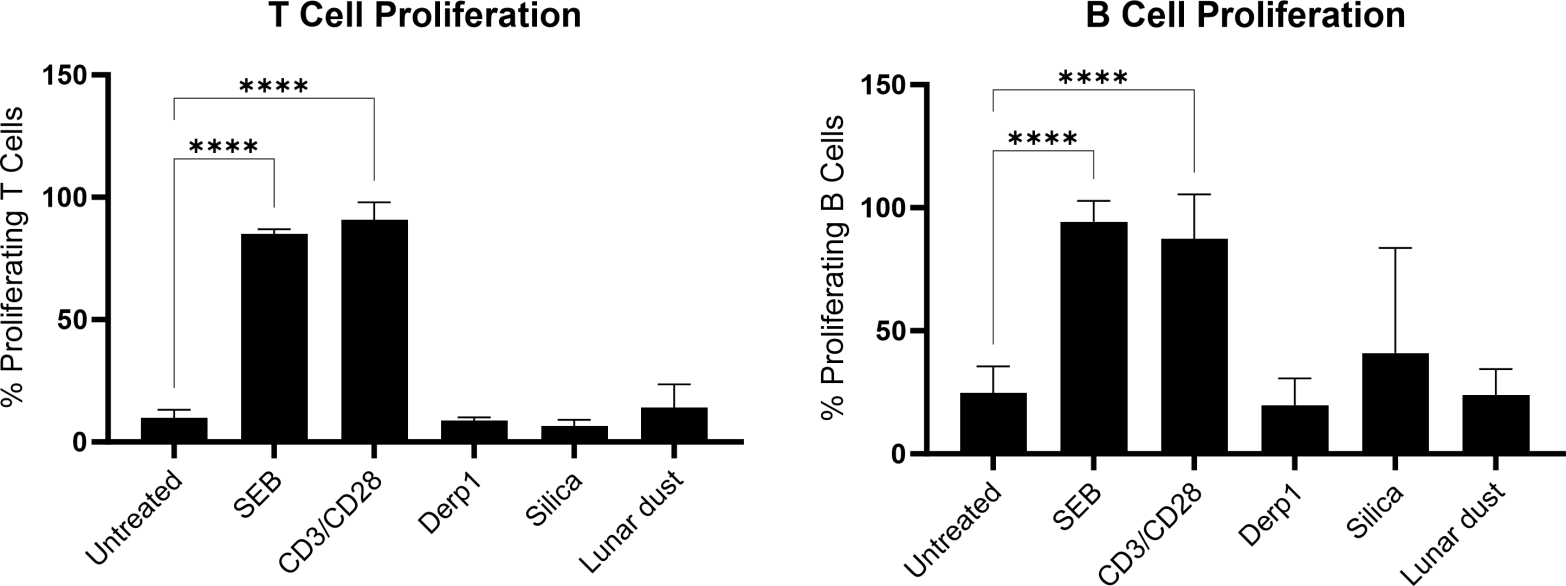
Percentage of **(A)** T cell and **(B)** B cell proliferation in 6 day cultures for each stimulation/treatment in comparison to untreated control cultures. **** P<0.0001.

In experimental setup, dying PBMCs with CFSE and culturing for 6 days, indicated that cell proliferation of T and B cells was not induced by Der p1, silica, or lunar dust, only the positive mitogenic controls, αCD3/αCD28 and SEB (**Supplementary Figure S3**). The percentage of T and B cells that proliferated were similarly significant (P>0.0001) for the positive controls (**Figure 12**). T cells are commonly induced for cell division during an immune response that leads to their activation (**Figure 3**), and B cells can be induced to proliferate as well in response to activated T cells producing the cytokine IL-4, which we confirmed is occurring in response to the mitogenic positive controls in our cytokine assay (**Figure 6**). T cell or B cell activation does not occur in response to Der p1, silica or lunar dust (**Figure 3**), which explains why proliferation does not occur either. This suggests that any irritation seen in crew in response to lunar dust is not a result of T or B cell immunoreactivity.

### 3.3 Microscopy analysis of cell-dust interactions

**Figure 13:**
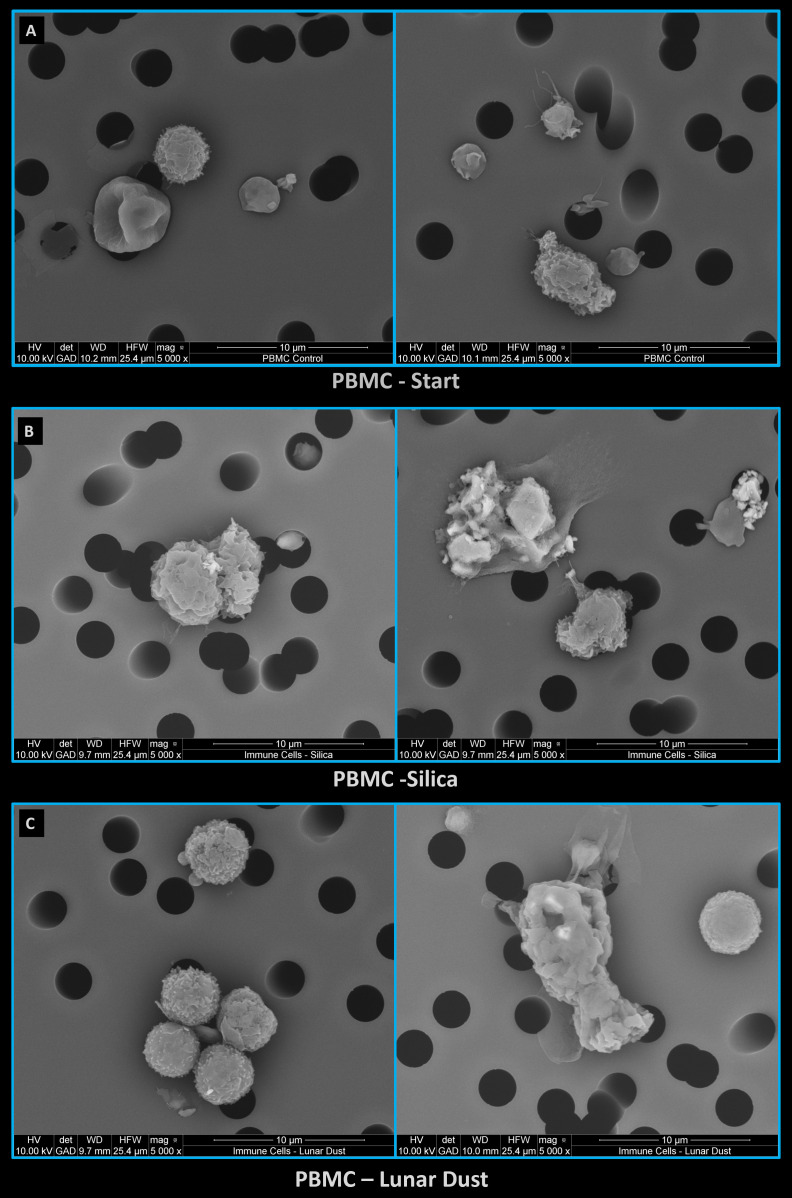
Representative images of PBMCs fixed following 48hr of culture with **(A)** control media, **(B)** media with silica, and **(C)** media with lunar dust.

To determine if *in vitro* exposure to lunar dust and silica (lunar dust simulant control) resulted in direct interaction of human peripheral blood immune cells, environmental scanning electron microscopy (ESEM) analysis was carried out on PBMCs from healthy human test subjects cultured with control media, media with silica and media with lunar dust. **Figure 13** shows representative images of PBMCs fixed following 48hr of culture with control media (**Figure 13A**), media with silica (**Figure 13B**) and media with lunar dust (**Figure 13C**). Image analysis was acquired using the environmental with a backscatter detector, which enables the detection of high energy particles such as silica and lunar dust, against the lower energy carbon-based cells. **Figure 13B**, left panel, shows silica sitting on top of the cell as depicted by the bright small material located at the center of the cells in the image while the right panel shows both a cluster of silica particles as well as an adjacent cell interacting with the silica. **Figure 13C** shows cells with a tiny lunar dust particle on their surface (left panel) which was the more common finding, while the right panel shows a representative cell that has ingested the lunar dust.

To better understand the size distribution of the specific lunar dust (sample ID 68501) that was used for coculture with the PBMCs, samples of the media with lunar dust that was utilized in the cell cocultures, was fixed for ESEM analysis then run through a particle size distribution macro using Image J. **Supplementary Figure S4A** shows a backscatter image of the lunar dust particles distributed through the sample while **Supplementary Figure S4B** shows the representative particle ID by number with the matching size distribution on the adjacent chart. Size distribution varied greatly with the smallest particle size at a surface area of 0.323μm2 (width and height of 0.744μm and 0.709μm) and the largest particle in this sample at a surface area of 60.3μm2 (width and height of 8.56μm and 10.4μm). **Supplementary Figures S4C, D** represent an even larger lunar dust particle isolated from the lunar dust-PBMC coculture sample that had a surface area measurement of 5760μm2 (width and height of 75.8μm and 111μm). Information on the Lunar Sample Compendium, show that lunar dust ID 68501 (https://curator.jsc.nasa.gov/lunar/lsc/68501.pdf) is a soil and rake sample collected from surface regolith of the ejector blanket of South Ray Crater at Station 8, with an average coarse grain size of 84μm – 115μm range. Although the size distribution varied greatly of the specific sample set we obtained, smaller grain sizes were detected which are more optimal for cell-particle interactions. For the subjects tested, there were limited cell-lunar dust interactions throughout the samples analyzed by ESEM. These results are similar to those described in the flow cytometry assessments. Despite limited cell-lunar dust interactions, **Supplementary Figure S5** shows a PBMC that has ingested a lunar dust particle (bright particle in cell detected by back scatter imaging). This confirms that for this lunar dust sample type, there is the occasional cell-lunar dust interaction when particulate size is optimal.

In addition to the imaging, energy-dispersive X-ray spectroscopy (EDS) analysis was carried out on the lunar dust and silica particles. It should be noted that due to the cellular composition of the samples, analysis could not be conducted above 10 KV in order to minimize destruction of the cells. This lower energy level was not sufficient to allow the detection of iron which is a classic hallmark for lunar dust. However, EDS analysis of lunar dust (**Supplementary Figure S6**, top panel) confirms that the particle present in the cell-lunar dust coculture was lunar dust. Similarly, EDS analysis of the cell-silica coculture also confirms the presence of silica. Last, EDS analysis of the bright spot detected on the cell of the lunar dust coculture sample confirms that the particulate ingested by the cell is lunar dust (**Supplementary Figure S6**, middle panel). This finding confirms that cells are able to ingest lunar dust.

## 4 Discussion

This study aimed to investigate the potential of immune reactions that are associated with clinical allergy to lunar dust primarily looking at the key immune cell responsiveness to lunar dust itself, a simulant, and other common recall antigens or polyclonal mitogens serving as positive controls. Human primary leukocytes isolated from blood were primarily used in this investigation. Eosinophils and basophils are known mediators of nasal allergic disease and make up less than 10% of the peripheral blood of healthy individuals so we also utilized two cell lines representing those populations. Cellular responsiveness to coculture in the presence of lunar dust was evaluated in several different ways. Our investigation assessed the bulk levels of key immune cells before and after culture by flow cytometry. The induction of cell surface activation markers was also determined by flow cytometry. Cytokines are small proteins produced by several cell types with many different functions. Some roles cytokines play is in cell signaling during an immune response to communicate to other immune cells for recruitment, expansion, activation or to produce more antigen specific cytokines. Not relying on cellular assessments, which can be difficult for activated cells, assaying supernatant cytokine profiles is an extremely sensitive and reliable indicator of both cellular activation and the specific nature of the response (inflammatory, Th1, Th2, etc.). There were no significant changes in cytokine production in response to the allergen, Derp1, or lunar dust. This indicates Derp1 and lunar dust do not induce an immediate and inflammatory immune response in this model. Allergic reactions are triggered by exposure to allergens, or usually harmless substances, such as dust, pollen, pet hair, etc., that induce the immune system into an immediate overactive Th2 inflammatory response by recruitment and expansion of cells, and production of cytokines, histamine, IgE, leukotrienes, etc. We measured soluble cytokines in culture supernatants by multiplex array and the production of allergy-specific products IgE, leukotriene B4, and histamine by ELISA. In any immune response to a foreign invader, the cascade of events for immune cells to respond after activation include rapid cell growth, or proliferation. T cells are a proliferating cell population in response to a foreign antigen or pathogen and this study used a cellular proliferation assay to determine if lunar dust induced this response. Flow cytometry was also applied to evaluate proliferative/blastogenesis responses of the T and B cell populations. Lastly, we employed microscopy to examine cell-lunar dust interactions.

Regarding the healthy subject primary immune cells, we did not find any significant changes in cellular activation or cytokine production in the human whole blood cultures in response to lunar dust. Allergy specific reactions indicated by increased histamine, leukotrienes, or IgE, were also not detected in subject whole blood cultures. Proliferative responses of any whole blood populations to lunar dust were not detected in any subjects.

For the cell line assessments, we did see direct activation of the eosinophils cell line (EOL-1) in response to lunar dust, however activation was also detected in response to silica (**Figure 5**). The response to silica and lunar dust in the eosinophil cell line cultures, but not in the primary whole blood cultures are likely due to a direct eosinophil response. Whole blood cultures have the benefits of all responding cell populations (not culturing artificially purified cells), and retaining soluble factors, resulting in a more *in-vivo*-like system. However, the other immune cells present that could mitigate responses from the eosinophil population, or more likely, the very low percentages of basophils and eosinophils in peripheral blood may have blunted any responsiveness. The basophil cell line (KU812) responded to lunar dust exposure by increased leukotriene B4 production (**Figure 9**) and the eosinophil cell line increased histamine after culture with lunar dust (**Figure 10**). No similar leukotriene or histamine responses were observed, in either cell line, to silica.

In short, assessments in primary human subject blood immune cells indicated no evidence whatsoever for sensitization, cellular responsiveness, nor ‘allergy’ to lunar dust. Possible caveats include the limited number of subjects used, the wide range of atopy seen in generally healthy individuals (the subjects were not known to be atopic) and a lack of previous sensitization (the subjects had not visited the lunar surface). During the Apollo reports of ‘allergy’ responses, those individuals similarly could not have been pre-sensitized, so we assume that any sensitization would have been via cross reactivity to some terrestrial product that induced cross reactivity to elicit an allergy-like response. Therefore, monitoring future Artemis crewmembers that will participate in lunar surface extravehicular activities for reactivity to lunar dust is appropriate to rule out some inherent existing reactivity to lunar dust.

Interestingly, assessments using purified ‘allergic’ cell lines, did yield some unique but mild responsiveness to lunar dust. At least for monitoring activation for the eosinophil cell line, responsiveness was observed to both lunar dust and silica. For leukotriene B4 and histamine, responses were seen that seem to be lunar dust specific. The eosinophil responsiveness to silica could indicate a manifestation of some sort of particulate response, however the leukotriene and histamine responses were more specific. While these results may seem disparate, for clinical interpretation we would prioritize the findings from the primary subject donor cell populations.

While no responses were observed from the test subjects, it should be disclosed that it is possible that these subjects, lacking prior sensitization to whatever terrestrial product that may induce immunogenic responses similar to lunar dust, were simply ‘not allergic’ to the product that lunar dust represents. The findings from the cell lines however, may represent the possibility that human allergenic cell sensitivity/reactivity to lunar dust is conceptually possible.

Future investigations could expand on this study. Certainly, an assessment of more human subjects with complete medical history data would allow a better determination of population sensitivity. More refined lunar dust with similar consistency in texture, size and granularity would also benefit the study. Lunar dust exposure is inevitable for future lunar exploration missions and efforts to reduce the amount of lunar dust exposure and countermeasures for addressing irritation will need to be put in to place. Our previous studies looking at the inhalation toxicity of lunar dust in rats (7), the immunoreactivity data of lung inflammation in response to inhalation of fine lunar dust particles and increased immune cell migration (17), and this study determining the allergy specific immune responses, will help guide NASA to develop mitigation techniques and potential countermeasures necessary in the event of excessive exposure to lunar dust during lunar surface EVAs.

## Data Availability

The original contributions presented in the study are included in the article/**Supplementary Material**. Further inquiries can be directed to the corresponding author.

## References

[1] Loftus DJ Rask JC McCrossin C Tranfield EM. The Chemical Reactivity of Lunar Dust: From Toxicity to Astrobiology. Earth Moon Planets. (2010) 107:95–105. doi: 10.1007/s11038-010-9376-x

[2] Scheuring RA Jones JA Novak JD Polk JD Gillis DB Schmid J. The Apollo Medical Operations Project: Recommendations to improve crew health and performance for future exploration missions and lunar surface operations. Acta Astronaut. (2008) 63:980–7. doi: 10.1016/j.actaastro.2007.12.065

[3] Crucian B Stowe RP Mehta S Quiriarte H Pierson D Sams C. Alterations in adaptive immunity persist during long-duration spaceflight. NPJ Microgravity. (2015) 1:15013. doi: 10.1038/npjmgrav.2015.13 28725716 PMC5515498

[4] Mehta SK Laudenslager ML Stowe RP Crucian BE Feiveson AH Sams CF. Latent virus reactivation in astronauts on the international space station. NPJ Microgravity. (2017) 3:11. doi: 10.1038/s41526-017-0015-y 28649633 PMC5445581

[5] Crucian B Johnston S Mehta S Stowe R Uchakin P Quiriarte H. A case of persistent skin rash and rhinitis with immune system dysregulation onboard the International Space Station. J Allergy Clin Immunol Pract. (2016) 4:759–762 e8. doi: 10.1016/j.jaip.2015.12.021 27036643

[6] Meyer C. (2010). Lunar sample compendium, in: The 41st Lunar Planetary Science Conference. TX, United States: The Woodlands. Available online at: https://ntrs.nasa.gov/api/citations/20090041867/downloads/20090041867.pdf.

[7] Lam CW Scully RR Zhang Y Renne RA Hunter RL McCluskey RA. Toxicity of lunar dust assessed in inhalation-exposed rats. Inhal Toxicol. (2013) 25:661–78. doi: 10.3109/08958378.2013.833660 PMC4666512 24102467

[8] IARC Working Group on the Evaluation of Carcinogenic Risks to Humans. Arsenic, Metals, Fibres and Dusts. Lyon (FR): International Agency for Research on Cancer; 2012. (IARC Monographs on the Evaluation of Carcinogenic Risks to Humans, No. 100C.) SILICA DUST, CRYSTALLINE, IN THE FORM OF QUARTZ OR CRISTOBALITE (2012). Available online at: https://www.ncbi.nlm.nih.gov/books/NBK304370/. PMC4781271 23189751

[9] Marshall GD Jr. Davis F. Dusting off recombinant allergens. Nat Biotechnol. (1997) 15:718–9. doi: 10.1038/nbt0897-718 9255778

[10] Marsman C Verhoeven D Koers J Rispens T Ten Brinke A. Optimized protocols for in-vitro T-cell-dependent and T-cell-independent activation for B-cell differentiation studies using limited cells. Front Immunol. (2022) 13:815449. doi: 10.3389/fimmu.2022.815449 35844625 PMC9278277

[11] Thyagarajan B Barcelo H Crimmins E Weir D Minnerath S Vivek S. Effect of delayed cell processing and cryopreservation on immunophenotyping in multicenter population studies. J Immunol Methods. (2018) 463:61–70. doi: 10.1016/j.jim.2018.09.007 30222961 PMC6423980

[12] Su KY Watanabe A Yeh CH Kelsoe G Kuraoka M. Efficient culture of human naive and memory B cells for use as APCs. J Immunol. (2016) 197:4163–76. doi: 10.4049/jimmunol.1502193 PMC5111638 27815447

[13] Bochner BS. Systemic activation of basophils and eosinophils: markers and consequences. J Allergy Clin Immunol. (2000) 106:S292–302. doi: 10.1067/mai.2000.110164 11080745

[14] Hirai K Miyamasu M Takaishi T Morita Y. Regulation of the function of eosinophils and basophils. Crit Rev Immunol. (1997) 17:325–52. doi: 10.1615/CritRevImmunol.v17.i3-4.40 9202886

[15] Yang S Chen L Zhang H Song Y Wang W Hu Z. Beyond the itch: the complex interplay of immune, neurological, and psychological factors in chronic urticaria. J Neuroinflammation. (2025) 22:75. doi: 10.1186/s12974-025-03397-4 40069822 PMC11895394

[16] Ogulur I Mitamura Y Yazici D Pat Y Ardicli S Li M. Type 2 immunity in allergic diseases. Cell Mol Immunol. (2025) 22:211–42. doi: 10.1038/s41423-025-01261-2 PMC11868591 39962262

[17] Crucian BE Quiriarte H Lam CW Nelman M Colorado AA Diak DM. Pulmonary and systemic immune alterations in rats exposed to airborne lunar dust. Front Immunol. (2025) 16:1538421. doi: 10.3389/fimmu.2025.1538421 39981230 PMC11840967

