# Supplementary figures and images for: Hazards of lunar surface exploration: determining the immunogenicity/allergenicity of lunar dust
Source: Front Immunol. 2025 May 8;16:1539163. doi: 10.3389/fimmu.2025.1539163 (PMC12094954; doi:10.3389/fimmu.2025.1539163)

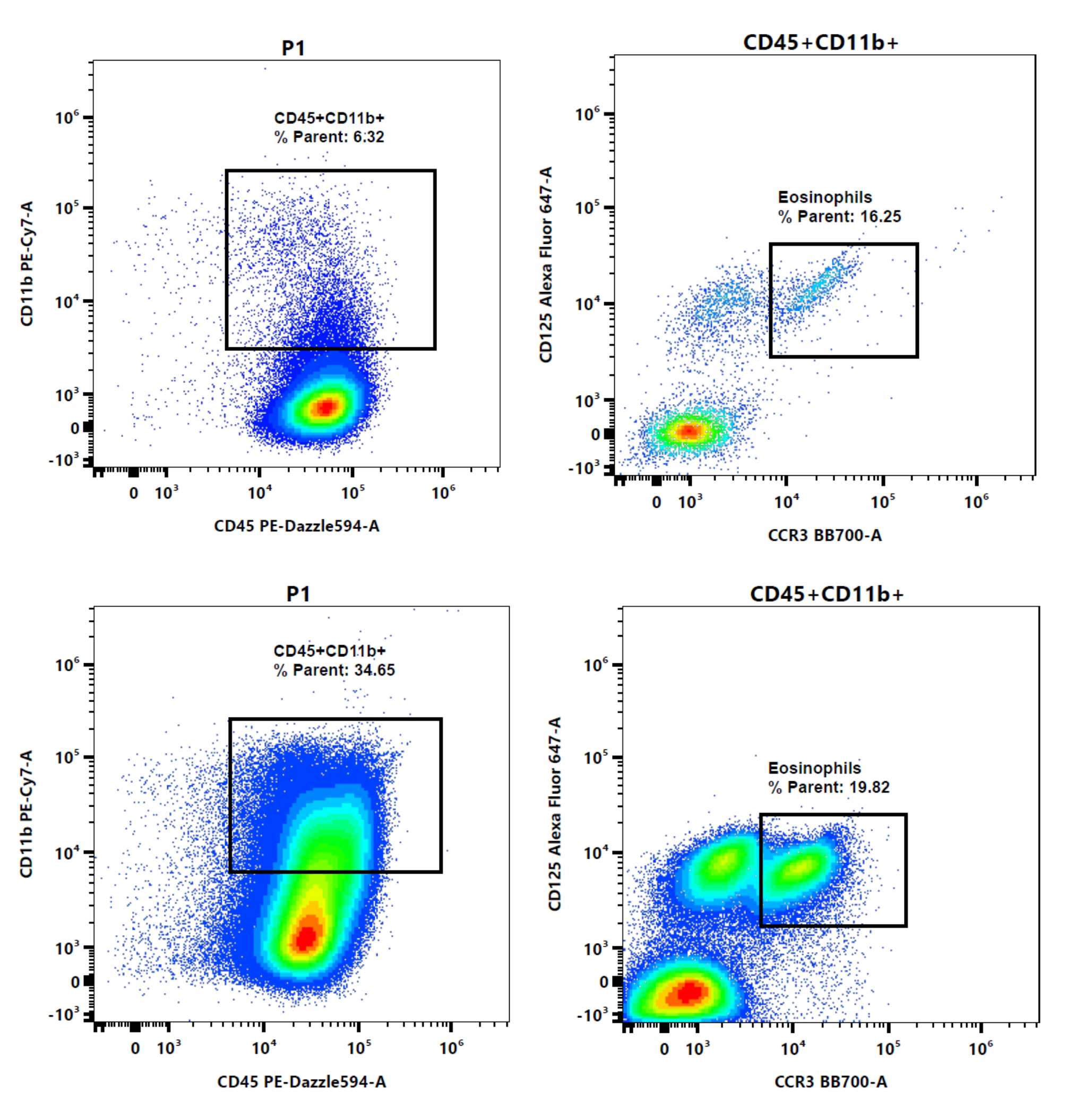

Supplement: Supplementary file 1 [file Image1.tif]

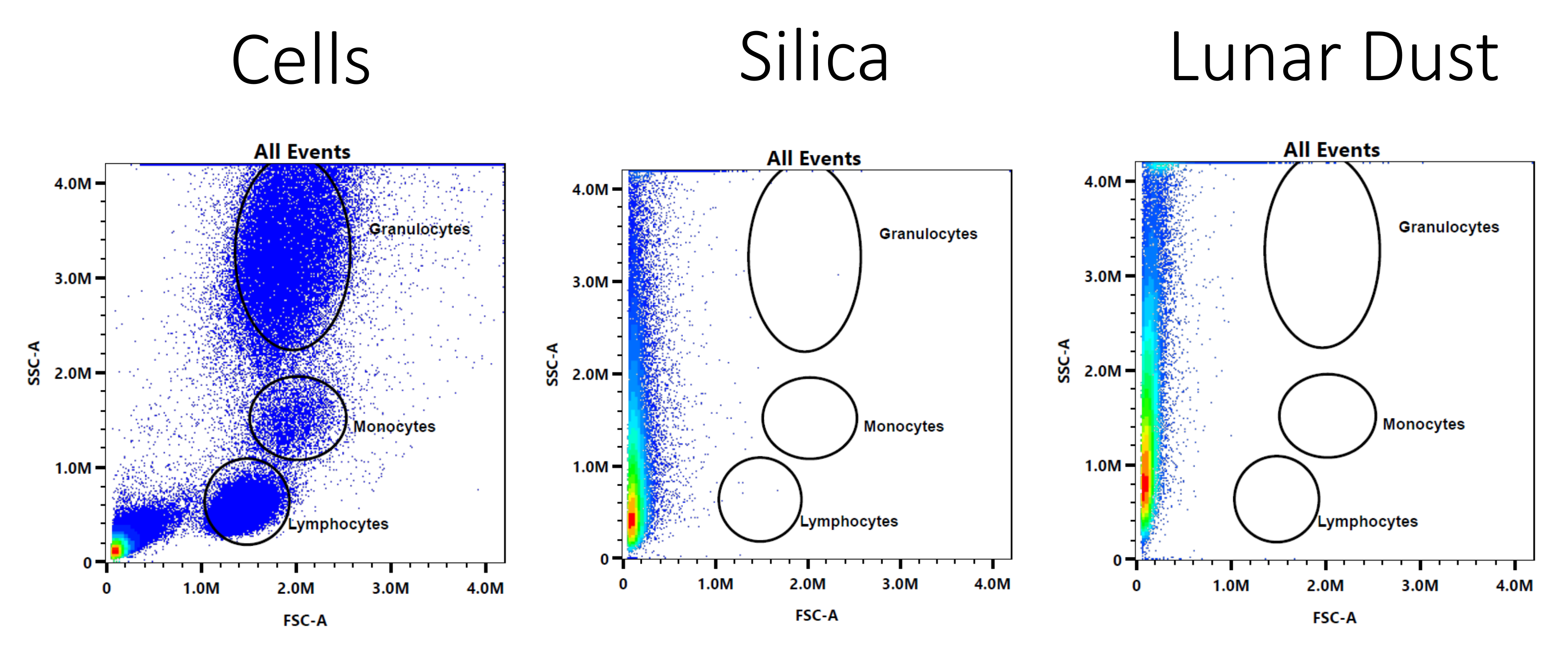

Supplement: Supplementary file 2 [file Image2.tif]

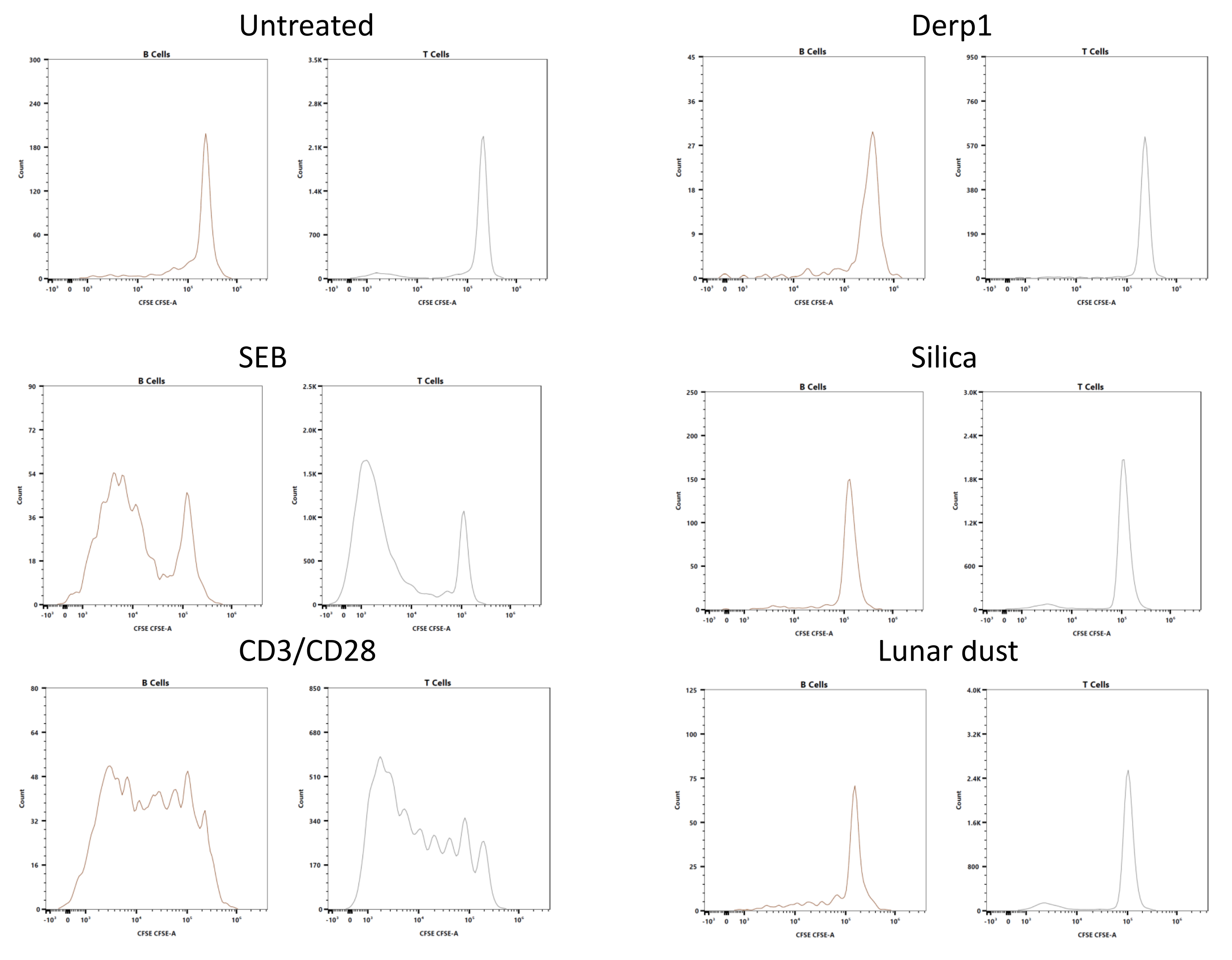

Supplement: Supplementary file 3 [file Image3.tif]

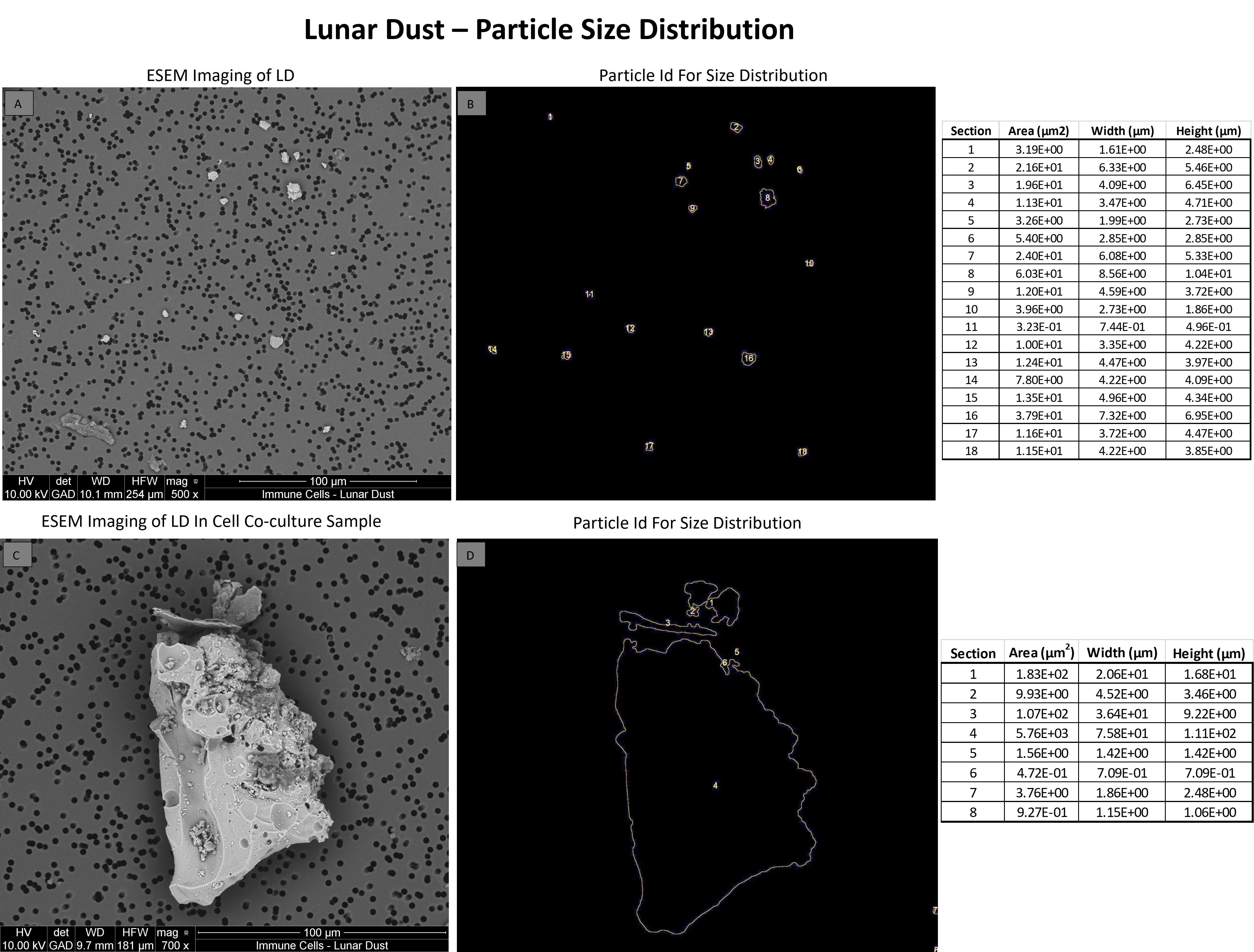

Supplement: Supplementary file 4 [file Image4.tif]

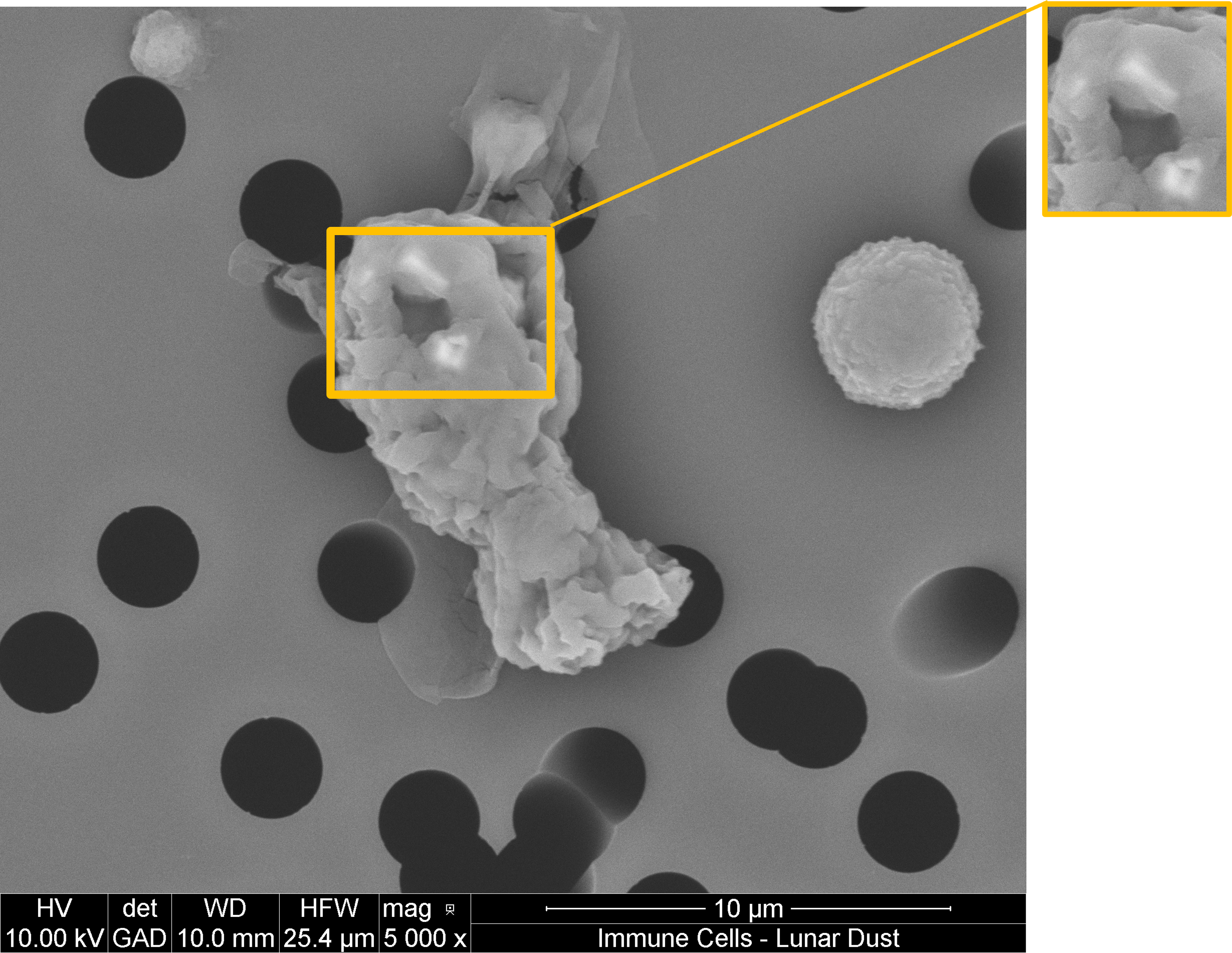

Supplement: Supplementary file 5 [file Image5.tif]

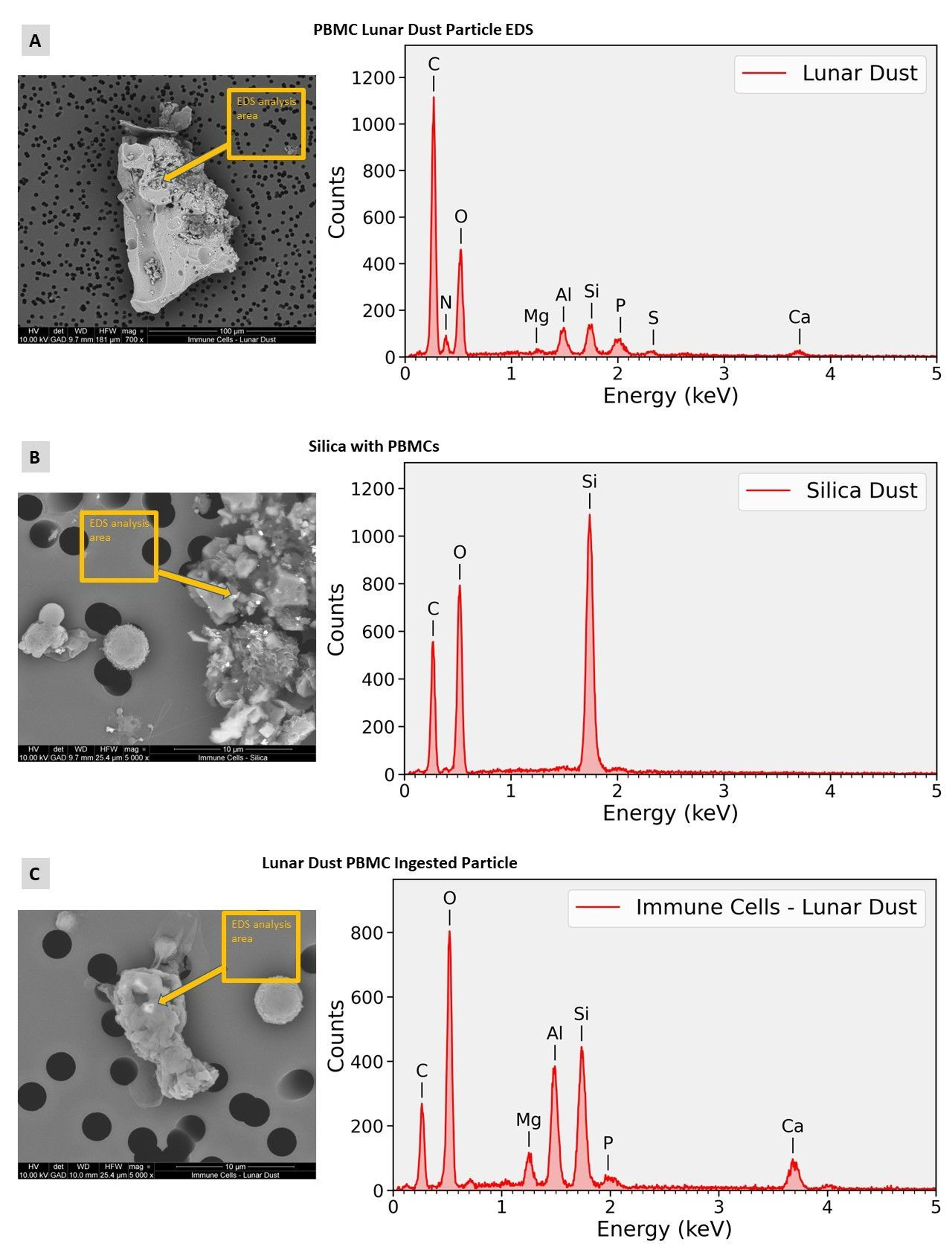

Supplement: Supplementary file 6 [file Image6.tif]
