# Supplementary material for: Hazards of lunar surface exploration: determining the immunogenicity/allergenicity of lunar dust
Source: Front Immunol. 2025 May 8;16:1539163. doi: 10.3389/fimmu.2025.1539163 (PMC12094954; doi:10.3389/fimmu.2025.1539163)
Supplement: Supplementary file 7 [file Table1.docx]

Supplementary Material

# Supplementary Figures and Tables

| **Marker** | **Cell Population** | **Fluorescent label** | **Manufacturer** |
| --- | --- | --- | --- |
| CD3 | T cells | BV510 | Biolegend |
| CD45 | Hematopoietic cells | PE/Dazzle 594 | Biolegend |
| CD19 | B cells | PeCy5.5 | Beckman Coulter |
| CD14 | Monocytes | BV605 | Biolegend |
| CD25 | T cell activation | FITC | BD Biosciences |
| CD69 | Activation marker | PE | BD Biosciences |
| HLA-DR | Activation marker | PerCP5.5 | Biolegend |
| CD125 | Eosinophils | AlexaFluor 647 | R&D |
| CD11b | Granulocytes | PECy7 | Biolegend |
| CCR3 | Eosinophils | BB700 | BD Biosciences |
| CD63 | Basophil activation | BV421 | Biolegend |
| CD123 | Basophils | PECy5 | Biolegend |
| CD203c | Basophil activation | BV510 | Biolegend |
| Ghost Dye | Viability stain | Ghost Red 710 | Tonbo |

Supplementary Table 1. Fluorescent antibody matrix for flow cytometry analysis

Supplementary Figure 1. Flow cytometry identifying eosinophil cells (CD45+CD11b+CCR3+CD125+) with and without differentiation treatment 5uM butyrate

Supplementary Figure 2. Forward and side scatter properties of silica and lunar dust compared to whole blood cellular populations.

Supplementary Figure 3. Flow cytometry histograms displaying generational cell division peaks of T cells and B cells for each treatment/stimulation in comparison to control.

Supplementary Figure 4. Lunar dust particle size distribution. (A) ESEM imaging of lunar dust and (B) particle identification for size distribution of lunar dust samples used in co-culture of immune cells. Size distribution ranges from surface area 0.323 μm2 with a width of 0.744 μm and height of 0.709 μm, up to a surface area of 60.3 μm2 with a width of 8.56 μm and height of 10.4 μm. (C) ESEM imaging of lunar dust in cell co-culture sample and (D) particle identification of lunar dust sample for size distribution of lunar dust particle that was in immune cell co-culture of confirmed cellular uptake. The large particle size had a surface area of 5760 μm2 with a width of 75.8 μm and height of 111 μm. Particles of smaller size distributions also present with the smallest particle size having a surface area of 0.472 μm2 with a width and length of 0.709 μm.

Supplementary Figure 5. Immune cell co-cultured with lunar dust contains a detectable high energy particle when imaged with a back scatter detector allowing for higher energy particles to be detected.

Supplementary Figure 6. Energy dispersive X-ray spectroscopy analysis of (A) lunar dust particle (B) PBMC lunar dust ingested particle and (C) silica co-cultured with PBMCs.
